# Are Corticosteroids Truly Contraindicated in Pulmonary Kaposi Sarcoma?

**DOI:** 10.7759/cureus.102101

**Published:** 2026-01-22

**Authors:** Sai Anoosh Parimi, Bryan K Dunn

**Affiliations:** 1 Pulmonary and Critical Care, East Carolina Medical Center, Greenville, USA; 2 Pulmonary and Critical Care, East Carolina University Brody School of Medicine, Greenville, USA

**Keywords:** anti-hiv agents, exudative pleural effusion, hiv aids, pulmonary kaposi sarcoma, systemic steroids

## Abstract

Kaposi sarcoma (KS) is an angioproliferative disorder associated with human herpes virus 8 (HHV-8) infection. It has both cutaneous and extracutaneous manifestations. With extracutaneous manifestations, it most commonly involves the oral cavity, gastrointestinal tract, and regional lymph nodes. Visceral organ involvement other than the gastrointestinal tract, such as the liver, lungs, and bones, is extremely rare. Here, we would like to discuss a patient with human immunodeficiency virus (HIV) and acquired immunodeficiency syndrome (AIDS) found to have pulmonary KS manifesting as acute hypoxemic respiratory failure (AHRF), along with bilateral pleural effusions and endobronchial lesions confirmed on bronchoscopy. He was treated with corticosteroids and paclitaxel, with significant improvement in AHRF. Traditionally, corticosteroids have been associated with exacerbation of pre-existing KS and even development of new-onset KS in HIV-infected patients and induction of KS. The experience gained from this case report suggests that judicious use of corticosteroids may improve the outcome of pulmonary dysfunction in HIV/AIDS-associated KS patients.

## Introduction

Pulmonary involvement of Kaposi sarcoma (KS) usually occurs in conjunction with extensive mucocutaneous disease [1,2]; however, in about 15% of cases, it can occur alone without mucocutaneous involvement [3]. Pulmonary manifestations include lesions involving the lung parenchyma, airways, pleura, and intrathoracic lymph nodes. Common clinical manifestations include dyspnea, hypoxemia, and progressively worsening cough associated with fevers and fatigue. Endobronchial obstruction can present with significant wheezing and atelectasis. Pleural effusions very occasionally occur as isolated manifestations. Adenopathy in pulmonary KS is of significant concern, as it can cause significant overt obstruction and can be a manifestation of opportunistic infections and secondary malignancies.

In the 1960s, the iatrogenic forms of KS were observed in solid organ transplant recipients, followed by the 1980s with the advent of the epidemic form of KS in young homosexual men in New York and California. Despite the prevalence of the disease for over 25 years, it is still one of the most frequent cancers in patients living with HIV. Human herpes virus 8 (HHV-8) as the causative agent for KS was established in 1994 after being isolated from patients with acquired immunodeficiency syndrome (AIDS) [3].

Historically, four forms of KS have been documented: classic (Mediterranean), endemic (African), epidemic (HIV/AIDS-associated), and iatrogenic (transplant-related). Its prevalence is substantially higher in men who have sex with men (MSM) and in certain regions of the world, such as sub-Saharan Africa or the countries bordering the Mediterranean, whereas it has been estimated to affect <5% of the general population of the USA and Europe [4].

In this case report, we would like to discuss a patient with a known diagnosis of KS associated with AIDS, who presented with acute hypoxemic respiratory failure (AHRF) and bilateral pleural effusions. He had prior mucocutaneous lesions, which had significantly improved while on antiretroviral therapy (ART) and doxorubicin. The patient was found to have endobronchial lesions consistent with KS on bronchoscopy and an exudative pleural effusion, and was treated with corticosteroids and paclitaxel. There was a significant improvement in his respiratory status during hospitalization, which led us to question whether corticosteroids may indeed be contraindicated in KS associated with HIV and AIDS.

## Case presentation

A 34-year-old male with a history of HIV and AIDS and prior diagnosis of KS of the oral cavity, and who was on ART with bictegravir, emtricitabine, and tenofovir alafenamide, was transferred to our institution for respiratory distress. According to the patient, he was having progressively worsening dyspnea, now occurring even during rest, and intermittent coughing spells associated with blood-tinged sputum, which progressed to frank hemoptysis.

The patient had been diagnosed with HIV four months prior to this presentation and was on ART. At the time of his diagnosis of HIV, he was also found to have oral lesions suggestive of KS and was started on doxorubicin. He had completed a total of six cycles, which led to significant improvement and resolution of his oral lesions. At the time of his initial HIV diagnosis, the oral lesions were accompanied by a persistent cough with blood-tinged sputum, and at that time, there was concern for pulmonary KS, but it was not readily diagnosed because, until the current episode, the above symptoms resolved after treatment with ART and chemotherapy. His cough and blood-tinged sputum had resolved until the current episode.

The patient was initially admitted at an outside hospital for a week prior to being transferred to our institution, where he underwent flexible bronchoscopy, which showed discrete areas of macular erythema throughout the tracheobronchial tree.

At that time, the bronchoalveolar fluid obtained from the right middle lobe was negative for Gram stain and did not grow any bacteria on cultures. Negativity was also observed for acid-fast bacilli stain and fungal stains, and cultures did not grow any fungal organisms. No evidence of Pneumocystis jirovecii pneumonia (PJP) was noted on PCR. On cytology, there were abundant hemosiderin-laden macrophages and no malignant cells.

**Figure 1:**
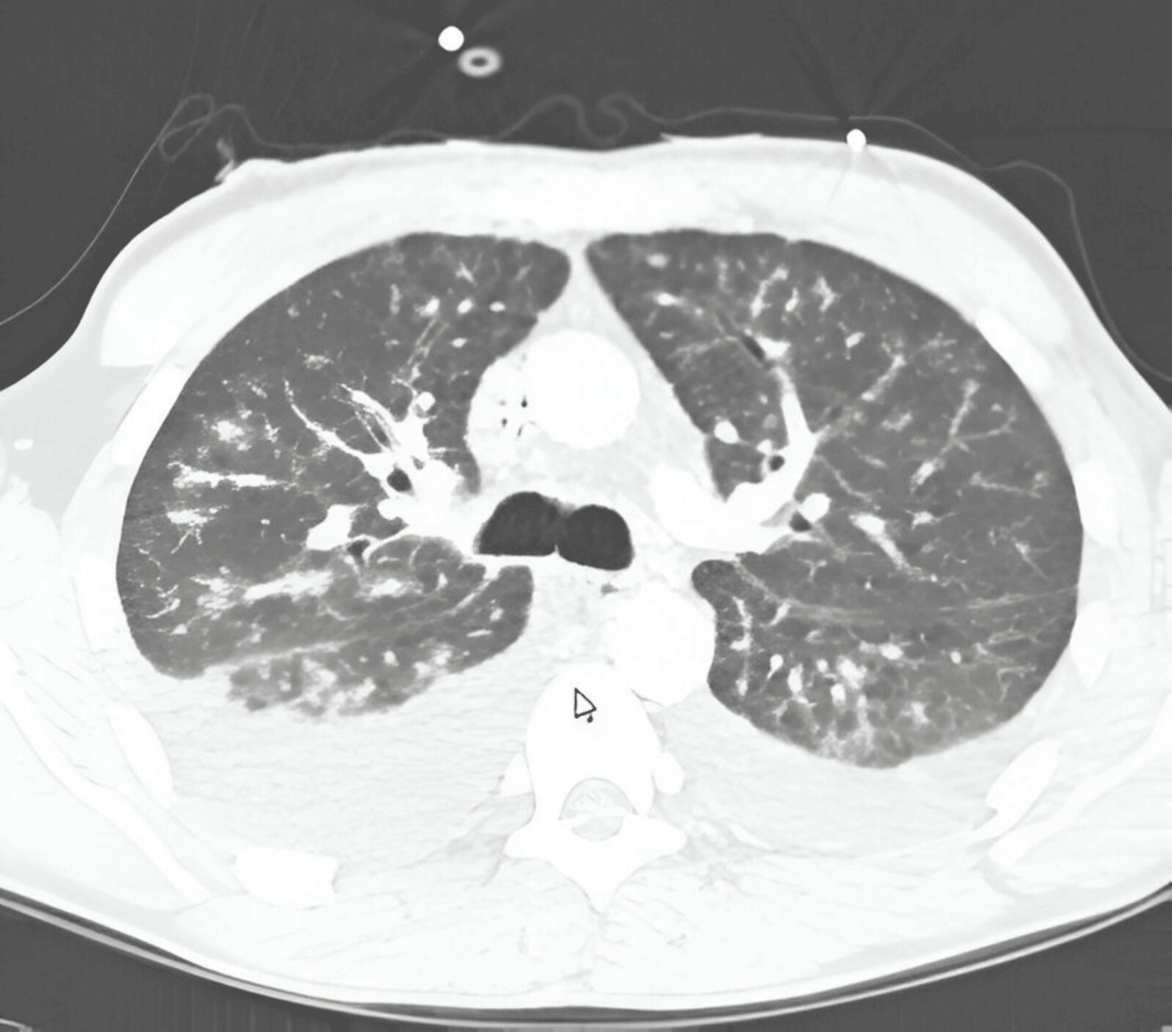
CT scan of the chest showing bilateral pleural effusions with patch consolidation.

Concurrently, he underwent a computed tomography (CT) scan with intravenous (IV) contrast of the chest, as seen in Figure 1, which showed no significant mediastinal and hilar adenopathy. There were irregular, diffuse, and bilateral nodular opacities, which were diffuse and bilateral with more lower lung predominance associated with significant interlobular septal thickening, along with fissure involvement, and bilateral ground-glass opacities. Also noted on the CT scan were small to moderate bilateral pleural effusions.

Despite being treated with broad-spectrum IV antibiotics, including vancomycin and cefepime, with trimethoprim and sulfamethoxazole for PJP, his hypoxemia deteriorated, and he was transferred to our institution for further management.

At our institution, he was found to be febrile to 101.6°F, hypoxemic with oxygen saturation (SpO2) of 94% on 60 L/min, and was placed on 100% fraction of inspired oxygen (FiO2) via high-flow nasal cannula.

**Figure 2:**
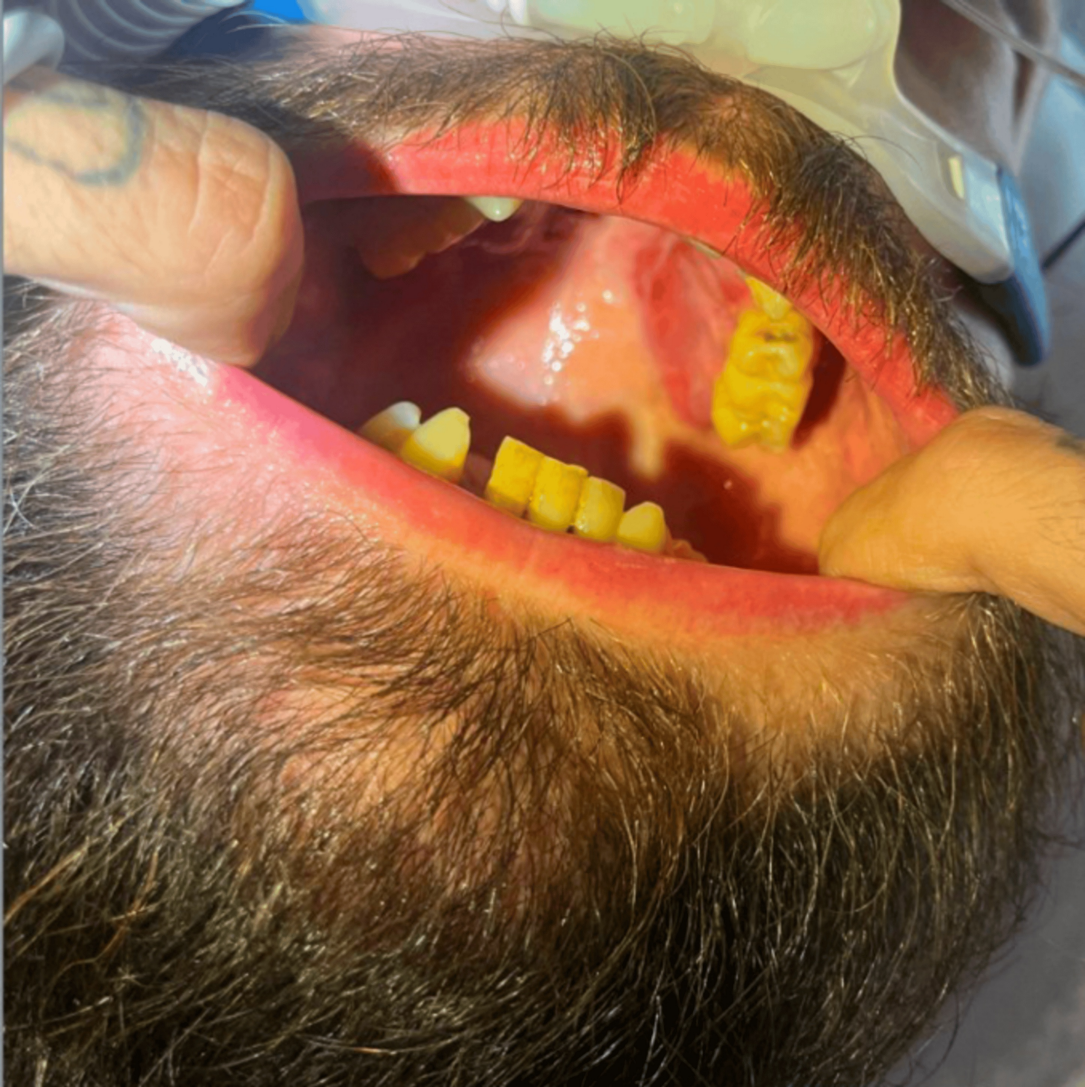
Oral examination revealing purple/red macules in the buccal cavity.

On examination, he was also found to have purple/red macules in the buccal cavity and bilateral papules in the lower extremities, as shown in Figure 2. No signs of cachexia, such as temporal wasting, were visible. No palpable supraclavicular, cervical, axillary, or inguinal adenopathy was noted.

**Figure 3:**
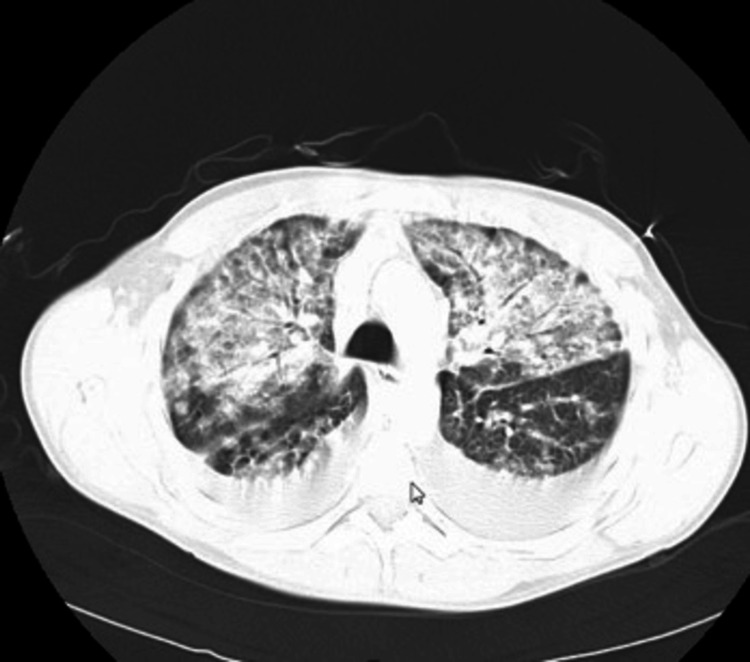
CT of the chest with IV contrast showing progression of the bilateral diffuse peribronchovascular and subpleural ground-glass opacities, with progression of consolidation. Bilateral small to moderate pleural effusions were noted, with unchanged mediastinal or hilar lymphadenopathy. No central airway obstruction was found.

The patient then underwent a second chest computed tomography (CT) scan with IV contrast (Figure 3), which showed bilateral diffuse peribronchovascular and subpleural ground-glass opacities, which had progressed from the scan done a week back at the outside facility. Again, noted were bilateral small to moderate pleural effusions, with no change in the degree of mediastinal or hilar lymphadenopathy, and no central airway obstruction was noted.

His HIV ribonucleic acid (RNA) load was 60 copies/ml (was down from 11784 copies/mL four months back when he was diagnosed with HIV), and his absolute CD4 count was 32 cells/mm^3^.

He underwent left-sided thoracentesis with 1000 cc of serosanguineous fluid. Biochemical analysis revealed the effusion to be exudative in nature, as shown in Table 1. There was no significant improvement in the degree of hypoxemia after the thoracentesis. The patient continued to be on 60 L/min 100% FiO2 via high-flow nasal cannula and humidified oxygen.

**Table 1:** Pleural fluid analysis.

Biochemical markers in pleural fluid	Value (Normal range)
Albumin fluid	2.4 g/dL (1-2 g/dl)
Glucose fluid	92 mg/dL (60mg/dl)
Lactate dehydrogenase (LDH) fluid	535 U/L (<50% plasma)
Total protein	3.9 g/dL (1-2 g/dl)
Total cholesterol	61 mg/dL (<45 mg/dl)
Pleural fluid cells - differential count	Cells/L (Normal range)
Total nucleated cells	219 (<1000 cells/mm^3^)
Red blood cells (RBC)	186,286 (<10000 cells/mm^3^)
Neutrophils	23% (1%)
Lymphocytes	64% (23%)
Monocytes	12%
Eosinophils	1% (0%)
Macrophages/histiocytes	Scattered (73%)
Mesothelial cells	Numerous (1-2%)
pH	7.8 (7.6-7.66)

Cytology of the pleural fluid was negative for malignancy and showed reactive mesothelial cells, with macrophages and lymphocytes. Stains for Pneumocystis and other fungal organisms were negative. Gram stain and cultures were negative for any bacteria, and no fungal organism growth was detected.

The patient had clearly expressed that he did not want to be intubated and hence was managed conservatively with intermittent bi-level positive airway pressure (BiPAP) and high-flow nasal cannula with humidified oxygen.

After discussing with our colleagues in infectious disease as well as oncology, he was continued on ART (bictegravir/emtricitabine/tenofovir alafenamide) and was started on paclitaxel (100 mg/m^2^), and received the first dose while inpatient. For his AHRF, he was treated with broad-spectrum antibiotics, including seven days of piperacillin and tazobactam, azithromycin 500 mg for three days, and trimethoprim and sulfamethoxazole for PJP prophylaxis. He was concurrently treated with corticosteroids, i.e., methylprednisolone 60 mg twice a day. Over a span of seven days, his respiratory status significantly improved, and he was thus transitioned from 60 L/min and 100% FiO2 to 6 L/min supplemental oxygen via nasal cannula.

## Discussion

KS is caused by an infection with HHV-8, which involves viral oncogenesis. Among HIV patients, the incidence and mortality associated with KS have been significantly reduced with the advent of ART. Although some case reports have found that administration of corticosteroids to HIV-infected patients was associated with worsening clinical outcomes [5-11], the evidence remains circumstantial, and there are no published studies to prove the same. This is likely due to the low incidence of KS in the antiretroviral drug era. Corticosteroids have always been used in HIV patients with severe PJP. In one retrospective study evaluating corticosteroids as a risk factor for KS-associated immune reconstitution inflammatory syndrome (IRIS), of the 145 patients who developed KS after being started on ART, 60 of these patients were on glucocorticoid therapy [11]. In the subgroup that had received glucocorticoids, the KS-associated mortality was statistically significant (P = 0.004, 95% CI: 1.727-18.1), and time to death was significantly shorter than that of the non-glucocorticoid-treated cases. However, a confounding factor here was KS-IRIS, which was in fact reported as a risk factor regardless of corticosteroid use. Also, these patients had an absolute CD4 count of <50 cells/microliter. Of note, glucocorticoids have been clinically associated with the development of KS in non-HIV patients such as transplant recipients [12-14], although such a correlation has not been strictly validated, either statistically or pathologically.

Corticosteroids are anti-inflammatory agents thought to act by suppressing the natural killer (NK) cell activity. Concern on the use of corticosteroids in cancer patients arise from their immunosuppressive effects, which seem to regulate tumor cell proliferation, which may impair immune surveillance, ultimately culminating in and facilitating tumor progression via oncogenesis. Further, glucocorticoids also seem to directly promote the proliferation of KS spindle cells by modulating glucocorticoid receptor expression [14] and HHV-8 activation [15].

## Conclusions

We report a patient who had presented with KS and was treated with a short course of glucocorticoids for pneumonia, after which improvement of his AHRF was observed. The medication did not worsen his short-term outcomes, as he was discharged home after AHRF resolution. While studies suggest a causal relationship between glucocorticoid use and KS development, it is worth noting that KS is only one of many causes of mortality in HIV/AIDS patients. Therefore, in clinical situations demanding a regular standard of care, corticosteroids must be used judiciously.
